# Supplementary material for: Assessing the Role of Ancestral Fragments and Selection Signatures by Whole-Genome Scanning in Dehong Humped Cattle at the China–Myanmar Border
Source: Biology (Basel). 2022 Sep 9;11(9):1331. doi: 10.3390/biology11091331 (PMC9495559; doi:10.3390/biology11091331)
Supplement: Supplementary file 1 [file biology-11-01331-s001.zip › Supplementary Figure S1.pdf]

A

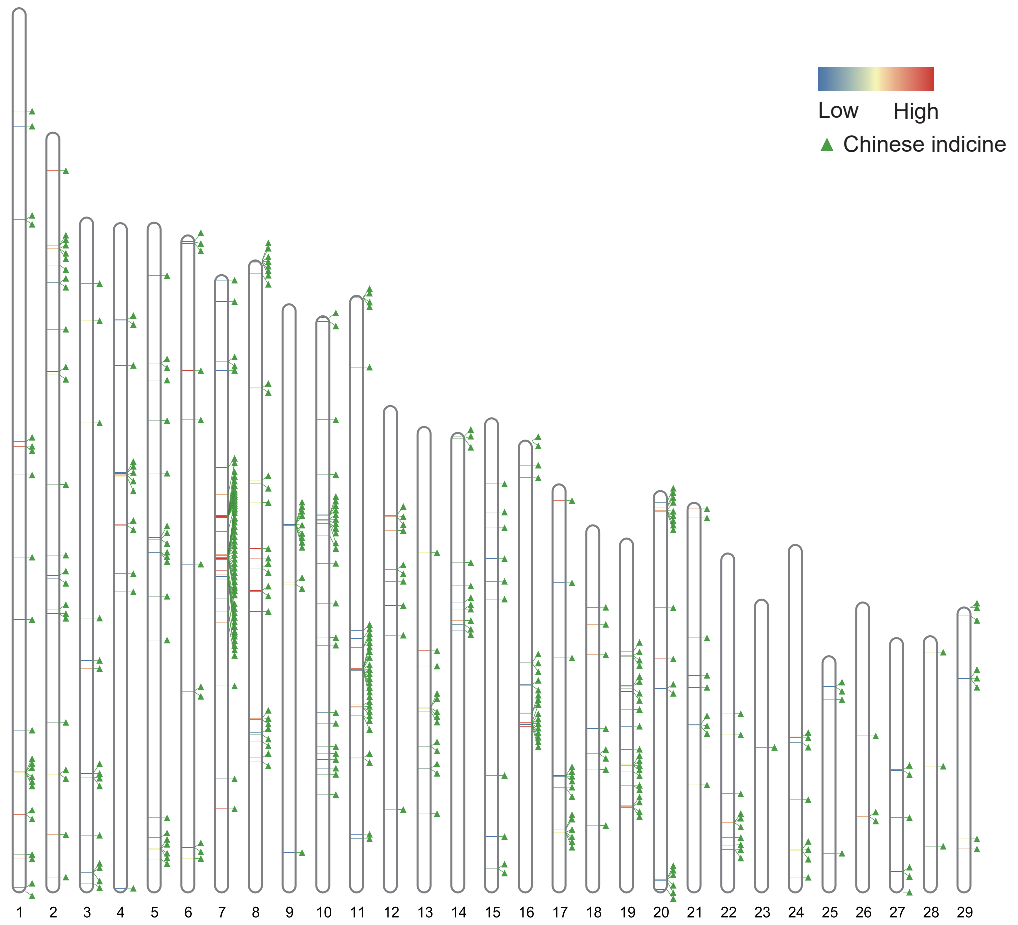

B

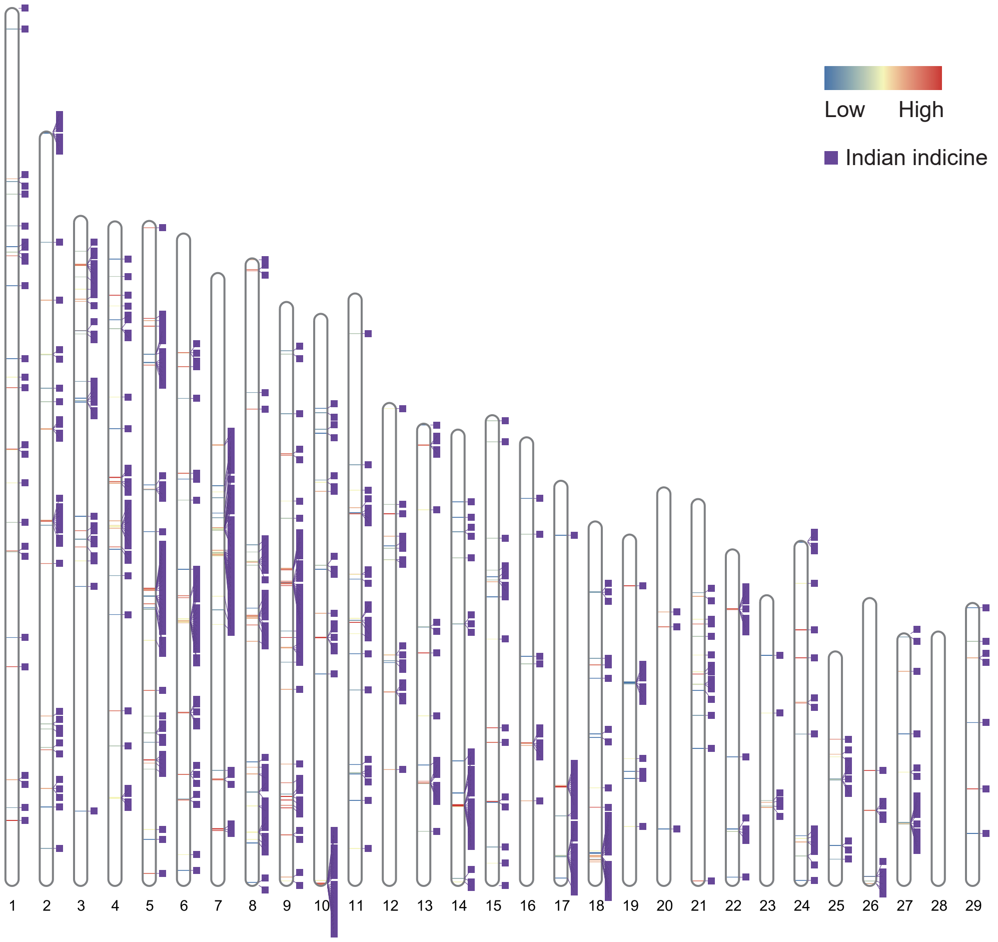

Supplementary Figure S1. (A)Distribution of the local segments whose proportions of Chinese indicine were excessive compared with the average level of whole genome. (B)Distribution of the local segments whose proportions of Indian indicine were excessive compared with the average level of whole genome.
